# Evaluation of testosterone serum levels in patients with obstructive sleep apnea syndrome

**DOI:** 10.1590/S1808-86942011000100015

**Published:** 2015-10-19

**Authors:** Fernando Drimel Molina, Marcela Suman, Thiago Bittencourt Ottoni de Carvalho, Vânia Belintani Piatto, Sebastião Roberto Taboga, José Victor Maniglia, Waldir Antônio Tognola

**Affiliations:** 1 Head of the Otorhinolaryngology and Head & Neck Surgery outpatient unit, FUNFARME/FAMERP; 2 Third-year medical resident in otorhinolaryngology and head & neck surgery, FAMERP; 3 Medical graduate, fourth-year medical resident in otorhinolaryngology and head & neck surgery, FAMERP; 4 Doctoral degree in health sciences, adjunct professor III-D of the Otorhinolaryngology and Head & Neck Surgery Department, FAMERP; 5 Associate professor, UNESP. Adjunct professor of the Biology Department, UNESP; 6 Associate professor, UNESP. Adjunct professor of the Otorhinolaryngology and Head & Neck Surgery, FAMERP; 7 Associate professor, graduation tutor, FAMERP

**Keywords:** blood cell count, sleep apnea obstructive, obesity, obstructive, cell hypoxia, testosterone

## Abstract

Males with obstructive sleep apnea syndrome (OSAS) may present decreased testosterone serum levels because of hypoxemia.

**Aim:** To correlate testosterone levels in OSAS patients with laboratory parameters.

**Material and methods:** 103 registries of OSAS patients were reviewed from 2002 to 2009. The following data collected: age when polysomnography was done, hematocrit and hemoglobin levels, total testosterone serum levels, BMI, apnea/hypopnea index (AHI), and O2 saturation.

**Study Design:** A cross-sectional retrospective case study.

**Results:** 79 patients (77%) had no hormonal changes, and 24 patients (23%) had decreased serum levels. In patients with normal testosterone levels, 70% were overweight; 63% with altered testosterone levels had obesity grade I (*p*<0.05). Patients with altered testosterone levels had significantly lower average doses of Ht, Hb and androgen compared to patients without altered androgen levels. The average BMI of patients with altered hormone levels was significantly higher compared to patients with normal hormone levels.

**Conclusions:** The relationship between morning testosterone levels and obesity, and to a lesser degree age, AHI and hypoxemia may be the cause of central suppression of testosterone in these patients. Decreased blood HT and HB levels may be related to lower levels of circulating testosterone.

## INTRODUCTION

The obstructive sleep apnea/hypopnea syndrome (OSAHS) is a common respiratory condition that affects about 4% of males and 2% of females; it may be characterized by recurring sleep-induced collapse of pharyngeal airways that result in hypoxemia and hypercapnia.^1^ Variables that cause pharyngeal collapse include negative airway pressure during inspiration and positive pressure on the outside of these airways because of fat deposits and/or small jaws.^2^

Although the underlying mechanisms of sleep apnea/hypopnea remain unknown, altered central nervous system control of upper airway muscles is an important component of this syndrome.^3^ The pathogenesis involves collapse of upper airways; for normal breathings, its patency depends on a specific dilator muscle. The genioglossus - one of the upper airway muscles - is the main extrinsic muscle for tongue protrusion; it is innervated by the hypoglossal nerve.^2,^^4^ The genioglossus contracts during inspiration, supporting lung ventilation and causing the upper airways to remain open. The central nervous system controls airway function from the nose to the bronchioles; it is an essential component of the breathing regulating system and airway patency during vigil and sleep. Serotoninergic and noradrenergic neurons - in parallel pathways - may directly stimulate the motor cells of the hypoglossal nerve; they regulate the upper airway dilator muscles. A higher activity of these neurons increases the activity of the genioglossus muscle.^4^

Age and the level of sex hormones affect the serotoninergic function. Serotonin production in neurons of the anterior horn of the cervical spine (associated with the motor nucleus of the phrenic nerve) decreases with age.^5^ There are several mechanisms by which estrogens elevate serotonin levels.^6^ Age-related changes in the serotoninergic system and their effect on respiratory motor control may occur indirectly by changes in gonadal hormone levels.^7^ The effects of age and sex hormones on responses to hypoxia have not been investigated in depth, in particular their impact on respiratory plasticity following intermittent hypoxia.^8,^^9^ This is of significant interest, as several respiratory conditions, including the OSAHS, appear to be related to age and gender.^10,^^11^

Aromatase converts testosterone into estradiol, which mediates most of the actions of testosterone on the serotoninergic system; thus, testosterone in itself does not account for the degree of long-term facilitation - a form of respiratory plasticity expressed as a persistent elevation of phrenic and hypoglossal nerves following intermittent hypoxia. Sex hormone serum levels reflect circulating levels - but not necessarily the level in respiration-associated areas of the central nervous system (caudal raphe and the phrenic and hypoglossal nerve nuclei). Aromatase is part of the group of p450 cytochrome enzymes; it is a mediator of aromatization, converting androgens into estrogens.^7^ Serotoninergic neurons in the caudal raphe nuclei send projections to the motor nuclei of the hypoglossal and phrenic nerves; there is evidence that sex hormones modulate respiratory plasticity through the serotoninergic system.

Depletion of gonadal hormones may decrease serotonin uptake in the hypoglossal and phrenic motor nuclei, thereby reducing long-term facilitation. Androgenic effects on the serotoninergic system appear to be indirect, reflecting testosterone conversion into estradiol in the central nervous system.^12^ Lower estradiol levels reduce serotonin synthesis, release, reuse and degradation; terminal serotonin levels and receptor density in several areas of the brain are also decreased. Thus, estradiol appears to have a key role in modulating serotoninergic function, thereby affecting long-term respiratory facilitation.^13^, ^14^, ^15^

OSAHS affects middle-aged men more often than women; thus, it is no surprise that testosterone is involved in respiratory control. Testosterone, estradiol and progesterone circulating levels decrease with age in men.^16^ A few effects of sex hormones in respiratory control are mediated by aromatase-mediated testosterone conversion into estradiol in the brain.^12^ However, it is not clear if aromatase declines sufficiently to reduce respiratory plasticity as patients age.^7^ Reports of male subjects with OSAHS and low testosterone levels - regardless of age - have been published.^17^

The purpose of this study was to assess quantitative serum testosterone levels in patients with OSAHS, and to relate these levels with age, body-mass index, the apnea/ hypopnea index, maximum oxygen saturation, the hematocrit (Ht), and hemoglobin (Hb).

## SERIES AND METHOD

A retrospective cross-sectional study was made of 103 registries of patients with a diagnosis of OSAHS from 2002 to 2009. The inclusion criteria were as follows: a) subjects that met criteria A or B and the items in criteria C, for diagnosis.

A. excessive daytime drowsiness not explained by other causes.
B. two or more of the following, not explained by other causes:
  gasping during sleep;
  recurring awakenings during sleep;
  restless sleep;
  daytime fatigue;
  difficulty to concentrate.
C. nighttime monitoring showing more than five episodes of respiratory obstruction per hour during sleep. These events may include any combination of obstructive apnea/hypopnea or respiratory effort related with awakening.
  b) total testosterone dosage in the morning and a red blood count following polysomnography.
  c) absence of other somatic or laboratory-identified disorders, as observed in the physical examination and work-up.
  d) absence of craniofacial dimorphism or temporomandibular disorders, as investigated in specific physical examinations.
  e) absence of drug dependency, alcoholism, depression, dementia, as investigated in a medical history and psychiatric assessment.
  f) absence of apparent genetic syndromes, as investigated by a medical-genetic physical examination.
  g) presence or not of other family cases.
  h) maximum age - 70 years.
  i) BMI ≤ 30.
  j) male gender.

The following data were gathered: age when undergoing polysomnography, Ht and Hb values, total testosterone serum level, body-mass index (BMI), apnea/ hypopnea index (AHI) and maximum O_2_ saturation - the last two based on polysomnography (Stellat System QC, Harmonie TM, Canada) recordings. Patients were allocated to the following age groups: young adults (18 to 40 years), adults (41 to 65 years), and elderly (> 65 years).^18,^^19^ The classification based on the apnea/hypopnea (AHI) was: mild OSAHS (AHI from 5 to 15 events/hour), moderate OSAHS (AHI from 16 to 30 events/hour), severe OSAHS (AHI > 30 events/hour).^20,^^21^ The classification of patients based on the BMI (World Health Organization) was: ideal weight (18.5-24.9 Kg/m^2^), overweight (25.0-29.9 Kg/m^2^), and grade I obesity (30.0-34.9 Kg/m^2^).^22^ A BMI ≤ 30 limit was established so that higher obesity grades would not bias serum testosterone levels.

A peripheral venous blood sample was taken in a dry tube to measure quantitative total testosterone by the competitive immunoassay method (direct chemiluminescent technology) using an ADVIA Centaur (Siemens Medical Solutions Diagnostics, NY, USA) commercial system. Peripheral venous blood was also sampled and placed in a tube containing EDTA anticoagulant for the red blood count. The Ht and Hb levels were measured with an automatic cell counter (Horiba ABX Diagnostics, Pentra DF 120, Montpellier, France). Reference values for total testosterone (245-1836 ng/dl), the Ht (40.7-50.3%), and Hb (14.5-18.0 g/dl) in men are aligned with the protocols of the cell counting and hormone dosage systems of the Central Laboratory of our institution.

The institutional review board approved this study (Document no. 365/2009).

### Statistics

The results were analyzed statistically for normalcy. The two-tailed Student's t test was applied for normally distributed independent samples, and the Mann-Whitney test was applied for non-normally distributed samples. The chi-square test was applied when appropriate for comparisons among variables. The significance level was 5%.

## RESULTS

Of 103 OSAHS patients, 79 (77%) had testosterone levels within normal limits, and 24 (23%) had testosterone levels below reference values.

Adults comprised 57 patients (72%) with normal testosterone levels and 18 patients (75%) with altered testosterone levels. No patient aged over 65 years had altered testosterone levels.

The BMI showed that 70% of OSAHS patients with normal testosterone levels were overweight, while 63% of OSAHS patients with altered testosterone levels had grade I obesity; this relation was statistically significant (*p*<0.05).

The AHI revealed that the severe grade was more prevalent in both OSAHS patients with normal (52%) and altered (50%) testosterone levels. Similarly, maximum O_2_ saturation over 90% was prevalent in both OSAHS patients with normal (84%) and altered testosterone levels (79%).

Table 1 shows the general data for the sample.

**Table 1:** Distribution as percentages (%) of the serum testosterone results found in OSAHS patients (n=103)

Variables	Normal testosterone 79 (77%)	Low testosterone 24 (23%)	p*
BMI			
Overweight	55 (70%)	9 (37%)	
Grade I obesity	24 (30%)	15 (63%)	<0,05
Age group (Classification)			
Young adult	21 (27%)	6 (25%)	
Adult	57 (72%)	18 (75%)	>0,05
Elderly	41 (52%)	0 (0%)	
Polysomnography (PSG) data			
AHI			
Mild	28 (35%)	5 (21%)	
Moderate	10 (13%)	7 (29%)	>0,05
Severe	41 (52%)	12 (50%)	
Maximum O2 Saturation			
> 90%	66 (84%)	19 (79%)	
< 90%	13 (16%)	15 (63%)	

AHI - apnea/hypopnea index.

BMI - body mass index.

Normal testosterone - serum levels from 245 to 1836 ng/dl.

Low testosterone - serum levels below 245 ng/dl.

* Chi-square test.

Table 2 shows that OSAHS patients with altered testosterone levels had significant lower mean Ht, Hb and serum testosterone levels compared to OSAHS patients with normal testosterone levels. The mean BMC of OSAHS patients with altered testosterone levels was significantly higher compared to the mean BMC of OSAHS patients with normal testosterone levels.

**Table 2:** Clinical and anthropometric features of OSAHS patients (n=103) and serum testosterone levels

Parameters	Normal Testosterone (n=79)	Low Testosterone (n=24)	*p*
Testosterone (ng/dl)	404,0 ± 132,6	200,4 ± 26,7	*p*≤0,001*
AHI (grade)	34,4 ± 24,4	36,1 ± 22,0	NS
Maximum O2 saturation (%)	91,6 ± 3,8	92,1 ± 4,3	NS
BMI (grade)	27,7 ± 2,3	29,4 ± 1,0	*p*≤0,01*
Age group (years)	46,1 ± 10,1	44,6 ± 9,8	NS
Hematocrit (%)	46,1 ± 3,5	44,4 ± 3,0	*p*≤0,05”
Hemoglobin (g/dl)	15,2 ± 1,1	14,6 ± 1,0	*p*≤0,05”

Values are presented as means ± standard deviation.

AHI - apnea/hypopnea index.

BMI - body mass index.

Normal testosterone - serum levels from 245 to 1836 ng/dl.

Altered testosterone - serum levels lower than 245 ng/dl.

* Mann-Whitney test.

“Two-tailed Student's t test for independent variables.

NS - Not significant

## DISCUSSION

There has been a growing awareness that sex hormones play an important role in nearly every physiological process, including breathing. Estrogen, progesterone, and testosterone may affect respiratory function in animals and humans.^9,^^23^ The prevalence of several respiratory disorders, such as OSAHS, the sudden death syndrome in infants, and Rett's syndrome, varies according to gender, which underlines the effect of sex hormones on breathing control.^24^, ^25^, ^26^

Long-term respiratory facilitation is an interesting model, making it possible to study the effects of sex hormones on neuroplasticity in general, and specifically on respiratory plasticity. Because of upper airway muscle rigidity, long-term facilitation allows breathing to become stable under conditions that otherwise might result in upper airway obstruction and apnea. Although the role of long-term facilitation has not been clearly defined as a causal factor of OSAHS, changes that affect respiratory plasticity - such as decreased sex hormone levels with aging - may foster the progression of this syndrome.^1^

The incidence of OSAHS is higher in men with lower serum testosterone levels, in middle-aged men,^27,^^28^ in women with low estrogen and progesterone levels, and in post-menopausal women not on hormone replacement therapy.^11,^^29,^^30^ The present study found low serum levels of total testosterone in OSAHS patients, most of which had a mean age of 44.6 years, grade I, and mild AHI; these results concur with other published studies.^17,^^27,^^31^, ^32^, ^33^, ^34^

Several factors may account for lower testosterone secretion levels in middle-aged men with OSAHS; these include: hypoxia, fragmented sleep, obesity, and advanced age. Previous studies on hypophysary-gonadal function in OSAHS have demonstrated that serum testosterone levels may be lower. Total and free testosterone levels were lower in obese males with OSAHS compared to age and body weight paired control group. A negative correlation was found between the severity of sleep apnea and testosterone levels; higher AHIs correlate with lower testosterone levels, suggesting that the severity of apnea was related with decreased testosterone secretion in OSAHS.^31,^^33^ This concept, however, remains controversial; lower testosterone levels have been found not only in patients with mild OSAHS but also in elderly obese patients with a severe AHI.^35^

Serum testosterone levels decrease with age. Hypogonadal testosterone levels have been found in about 12% of men aged over 50 years, and in 50% of men aged over 80 years.^27,^^36^ Central (hypophysary) and peripheral (testicular) effects cause age-related reduction in testosterone levels.^27,^^37^ In the present study, sub-normal hormone levels were found in 50% of severe OSAHS patients grave with grade I obesity (63%); however, most of these patients were in the adult age group (75%) rather than elderly subjects. Among patients with normal testosterone levels, the majority (52%) had severe AHI, and 72% were adults. The BMI placed these patients in the overweight group (70%).

Although published papers have reported an association between lower testosterone levels and higher than grade I BMIs^27,^^31^ our results in the present study showed that patients with lower testosterone levels and grade I BMI presented this association at a higher rate compared to patients with normal testosterone levels. This finding suggests that in patients with altered hormone levels, grade I BMI correlated with lower testosterone levels. Obesity is common in patients with OSAHS, and is associated with increased severity of sleep apnea, as shown by the AHI.^38^

Possible mechanisms to explain the association between OSAHS and obesity include several endocrine abnormalities involved in fat accumulation - especially abdominal fat - where lower androgen levels appear to have a significant role. Androgens stimulate fat lipolysis and inhibit the lipase lipoprotein, especially in the abdomen. Testosterone regulation of fat metabolism may explain why hypogonadal men invariably present increased levels of abdominal fat. Total and free testosterone levels fall in obese males - as body weight and fat increase - because of decreased globulin-bound hormone synthesis and altered activity of the aromatase system in peripheral tissues.^39^, ^40^, ^41^

Prior studies have shown that serum levels of testosterone are inversely correlated with the severity of OSAHS (AHIs); such altered levels result from fragmented sleep and decreased oxygenation, which are known inhibitors of testosterone production.^27,^^33^ A cross-sectional study of 225 male subjects undergoing polysomnography showed that the severity of OSAHS correlated significantly with globulin-bound sex hormones, decreased levels of total testosterone, and free testosterone.^42^ Our data did not confirm these results, as although 50% of OSAHS patients with low testosterone levels had severe apnea, a similar prevalence (52%) was found in OSAHS patients with normal testosterone levels. Further studies are needed to clarify which factors cause apnea index severity in the study population.

Oxygen desaturation affects testosterone levels by mechanisms that remain unclear. Decreased O_2_ availability during sleep has been associated with central inhibition of the hypothalamic-*hypophyseal-gonadal axis*, probably by increasing central endorphin levels. Furthermore, when patients are repeatedly exposed to upper airway obstruction episodes with clear sleep fragmentation, the circadian rhythm of testosterone appears to be affected.^43^ Prior studies have described the relative importance of hypoxia and fragmented sleep on the genesis of gonadal dysfunction; these studies have shown that sleep deprivation was associated with gonadal steroid suppression in normal young adults.^43,^^44^ Comparing OSAHS patients presenting severe O_2_ desaturation with OSAHS patients presenting less severe O_2_ desaturation revealed a significant correlation between peak testosterone levels and the total O_2_ desaturation time. This finding suggests that hypoxia affected the circadian rhythm of testosterone, resulting in decreased morning levels of this hormone.^43,^^45^

OSAHS patients presenting chronic hypoxemia may have ventilatory issues, as hypoxemia affects the synthesis and activity of several neurotransmitters, thereby changing the function of central and peripheral chemoreceptors that control ventilation.^46^ Prior studies have demonstrated the effect of the AHI on testosterone secretion, and have emphasized the degree of hypoxia. It is possible that hypoxia and fragmented sleep affect the hypophysary-gonadal function in OSAHS patients. A low testosterone level and its significant association with the AHI suggest that gonadal dysfunction is a consequence of OSAHS, rather than an independent primary condition of the hypothalamic-*hypophyseal-gonadal axis*.^27,^^43,^^45^

Although the relation between patient groups and mean AHI values and mean PaO2 values was not significant in the present study, 63% of patients with low testosterone levels had a PaO_2_ < 90% compared to 16% of patients with normal testosterone levels. And although the mean Ht and Hb values were within normal limits, there was a statistically significant difference between both groups of OSAHS patients, indicating a relative decrease in these values in patients with lower testosterone levels.

Endogenous androgens in mammals stimulate erythropoiesis, raise the reticulocyte count and the number of blood cells, and promote erythropoietic activity in the bone marrow; castration has the opposite effect. Testosterone deficiency causes a 10% to 20% reduction in the Hb concentration, which may result in anemia. Young male subjects with hypogonadism generally have low red blood cell counts and lower levels of Hb compared to similarly aged males with normal androgen levels. Androgens affect normal hematopoiesis by directly stimulating - through testosterone - renal production of erythropoietin.^47^ Androgen deprivation in men reduces erythropoiesis; hypogonadism is frequently associated with decreased Ht levels, as in our sample, which however were still within normal reference limits. Age-related anemia is at least partially due to declining levels of circulating androgens in older men.^48,^^49^

OSAHS patients may have decreased testosterone levels probably because of a combined effect of fragmented sleep and hypoxia. As sleep begins, the plasma testosterone concentration is low because maximal levels are reached during the first hours of the morning. Higher testosterone levels at night appear to be associated with the first episode of REM sleep and REM/non-REM cycles. Peak testosterone levels coincide with the onset of REM sleep. Furthermore, sleep-related elevation of testosterone levels in young men is associated with the first episode of REM sleep at night. A study of fragmented sleep in young males showed that the nocturnal peak concentration of testosterone was lower in men that did not enter REM sleep.^45,^^50^ Thus, we chose to measure testosterone levels in the morning after polysomnography was done, thereby identifying nighttime increases or not of testosterone in patients.

Low serum testosterone levels have been noted in obese, diabetic, hyperlipidemic, and insulin-resistant patients, as well as those with the metabolic syndrome. Serum testosterone levels in men deserve additional studies because of advances in knowledge about the cardiovascular aspects of hypogonadism and about OSAHS with or without associated metabolic conditions. It is not yet clear if OSAHS results in decreased testosterone levels by itself, or if obese, insulin-resistant and/or diabetic older OSAHS patients are at a higher risk of having lower levels of testosterone.^17^

Our findings in the present study and other published results may have practical implications for the treatment of obese OSAHS patients. Nutritional and behavioral therapy for rapid weight loss and long-term maintenance of weight has an important role for improving respiratory function and reducing metabolic disorders. Furthermore, testosterone levels tend to increase significantly after weight loss, which may help keep weight within normal limits.^51^

Endocrine abnormalities in OSAHS may be reverted following three months of *continuous positive airway pressure (C*PAP) therapy.^28,^^32^ However, normal testosterone levels have also been found in OSAHS patients using CPAP or not,^52^ as encountered the present study. Differences in these results may be due to the degree of OSAHS, to globulin-bound sex hormone levels, or to intermittent duration of CPAP and other similar therapies; these situations may explain unaltered testosterone levels.

The data suggest that OSAHS in middle-aged men is associated with decrease androgen secretion, together with obesity and aging. Hypoxia and fragmented sleep may be additional factors that decrease testosterone levels in these patients,^33^ as we found in the present study. Testosterone has been an object of research and reviews in several studies of male subjects; few of these papers have touched on the interaction between testosterone levels and sleep. Additional knowledge about this relationship is important for understanding the basic physiology of sleep and to help discover the causes of specific health issues associated with sleep disorders, aging, and even working hours. Knowledge about the interactions between hormones and breathing opens new perspectives in drug therapies for sleep disorders; it also stimulates research about how the endocrine system affects breathing in health and disease.^53^

## CONCLUSION

OSAHS may be associated with decreased hypophysary-gonadal function in the study sample.

A relation between serum levels of testosterone in the morning and obesity may account for central testosterone suppression in the study sample.

No conclusion could be drawn from the study results about any relation between altered serum testosterone levels and age, the AHI, or hypoxia.

Decreased red blood cell hematimetric values in the study sample may be related with low circulating testosterone levels.
